# Bibliographical Notices

**Published:** 1846-02

**Authors:** 


*Elements of Pathological Anatomy; illustrated by colored engravings, and two hundred and fifty wood cuts.* By SAMUEL D. GROSS, M. D., Professor of Surgery in the Medical Institute of Louisville; late Professor of Pathological Anatomy in the Medical Department of the Cincinnati College; Surgeon to the Louisville Marine Hospital, etc. etc. Second Edition, thoroughly revised, and greatly enlarged. Royal 8vo. pp. 822. Ed. Barrington & Geo. D. Haswell. Philadelphia, 1845.

Since the days of Morgagni, the importance of pathological anatomy as a department of medical science has been acknowledged by every one. It is true that, without a proper observance of the phenomena of disease as manifested during life, the mere inspection of the lesions discoverable after death, would subserve no useful purpose. To become good therapeutists, we must first learn healthy anatomy, in connexion with physiology; we shall then be prepared to engage in the study of Semeiology, and its relations to morbid changes of structure. Notwithstanding the occurrence of disease and even of death, in numerous instances, without any change of structure appreciable during life or discoverable after death, it is not unreasonable to presume that material modifications do exist, and that the improvements going on in our science will hereafter enable us to detect them under circumstances in which they now elude our observation. Certain it is, that within a few years past, numerous and most important discoveries have been made in this department by the aid of the microscope, both as it regards the natural structure and offices of the solid and fluid parts of the body, and the altered conditions which occur in disease, and we may fairly presume that much more will yet be attained. The study and more general employment, too, of auscultation and percussion, in discovering the physical signs of disease, leads to the detection of structural changes in various organs of the body during life, under circumstances where no such alterations would formerly have been suspected. By these several means, aided by the improved state of chemistry, valuable discoveries have of late years been made in this branch of our science. Many of these, however, exist as isolated observations, scattered over the pages of the periodicals of the day. The task of collecting and arranging them, so as to present all that is known, under appropriate heads, and in a manner accessible to every one, is one of great labour but of the utmost utility. The work of Dr. Gross originally appeared in the year 1839; and that a second edition should now be called for, considering the size and cost of the work, and on a subject which comparatively few practitioners are sufficiently industrious and painstaking to investigate, is certainly flattering to him.

The work is divided into two parts: 1st. General principles of Pathological Anatomy. 2d. Special Pathological Anatomy. In the first of these divisions the alterations and products of morbid action are considered, as they occur in all the tissues, as *inflammation, effusion of serum, lymphization, suppuration, hæmorrhage, softening, gangrene, ulceration,* &c. &c. In the second part, the alterations of structure and condition, as observed in the several parts or systems of the body, are described, as *the blood, cellular texture, adipous texture, muscular system, arteries, veins,* &c. &c.

Although we do not find, even in this later edition, all the valuable observations supplied by recent chemical and microscopical investigations, sufficient is given to afford to the inquirer a very good general knowledge of the present state of the science. We mean no disparagement by this remark. The labours of physiologists, with the aid of chemistry and the microscope, are so unremitting and so prolific, that a day scarcely elapses without some useful discovery; and it would be no easy task to collect and embody the whole, scattered as they are, up to the time of publication. Nor has the author manifested any want of diligence, or a desire to avoid labour in the preparation of the present edition of his work. It "has been thoroughly revised, and an amount of new matter, equal to three hundred pages of the original, has been introduced. The number of illustrations has been increased from ninetyseven to nearly two hundred and fifty. Of these, about one-third are original."

The mechanical execution of the book, including the illustrations, is good. Even the coloring of some of the plates, contrary to what we see in most American publications of the kind, is creditable.

---

*A Popular Treatise on the Teeth; embracing a description of their structure, the diseases to which they are subject, and their treatment, both for the prevention and cure of those diseases; together with an account of the usual methods of inserting Artificial Teeth.* By Robert Arthur, Doctor in Dental Surgery, &c. 12mo. pp. 187. E. Ferrett & Co. New York, 1845.

We have not discovered in this work anything particularly new or different from what is contained in the more elaborate treatises on Dental Surgery; nevertheless, it appears to be a very good manual on the subject. It is written in a popular style, without the use of many technical terms, and may be read with advantage by those who have not the time or disposition to study the larger works.

